# Zwitterionic Cysteine Drug Coating Influence in Functionalization of Implantable Ti50Zr Alloy for Antibacterial, Biocompatibility and Stability Properties

**DOI:** 10.3390/pharmaceutics10040220

**Published:** 2018-11-08

**Authors:** Ioana Demetrescu, Cristina Dumitriu, Georgeta Totea, Cristina I. Nica, Anca Dinischiotu, Daniela Ionita

**Affiliations:** 1 Faculty of Applied Chemistry and Materials Science POLITEHNICAof Bucharest, Romania Str. Polizu1-7, 011061 Bucharest, Romania; i_demetrescu@chim.upb.ro (I.D.); dumitriu.cristina.o@gmail.com (C.D.); 2 Faculty of Biomedical Engineering POLITEHNICA of Bucharest, Romania Str. Polizu1-7, 011061 Bucharest, Romania; 3 Academy of Romanian Scientists, Spaiul Independentei 54, 050094 Bucharest, Romania; 4 Buftea, M. Burghele Hospital, Studiolului 5, 070000 Buftea, Romania; georgeta.totea@gmail.com; 5 Department of Biochemistry and Molecular Biology, University of Bucharest, 91-95 Spl. Independentei, 050095 Bucharest, Romania; cristina.nica@drd.unibuc.ro (C.I.N.); anca.dinischiotu@bio.unibuc.ro (A.D.)

**Keywords:** zwitterionic drug, cysteine, antibacterial effect, biocompatibility, stability

## Abstract

The present paper aims atincreasing the bioperformance of implantable Ti50Zr alloy using zwitterionic cysteine drug coating. Aspects such as stability, biocompatibility, and antibacterial effects were investigated with the help of various methods such as infrared spectroscopy (FT-IR), scanning electronic microscopy (SEM), electrochemical methods, contact angle determinations and cell response. The experimental data of zwitterionic cysteine coating indicate the existence of a hydration layer due to hydrophilic groups evidenced in FT-IR which is responsible for the decrease of contact angle and antibacterial capabilities. The electrochemical stability was evaluatedbased on Tafel plots and electrochemical impedance spectroscopy (EIS). The cell response to cysteine was determined with gingival fibroblasts measuring lactate dehydrogenase (LDH) activity, concentrations of nitric oxide (NO) and intracellular level of reactive oxygen species (ROS). All experimental results supported the increase of stability and better cells response of implantable Ti50Zr alloy coated with zwitterionic cysteine drug. The antibacterial index was measured against *Staphylococcus aureus* and *Escherichia coli*. It was demonstrated that the coating enhanced the production of intracellular ROS in time, which subsequently caused a significant increase in antibacterial index.

## 1. Introduction

Rising bioperformance of implantable materials involves various surface modifications improving stability, biocompatibility, antibacterial effect, and hemocompatibility [1,2,3], and is a continuous issue in tissue engineering, bioelectronics and drug delivery [4]. In the last years, metallic biomaterials such as Ti have been most often subjected to such modifications via various procedures [5,6] involving coatings with different drugs [7]. Since Ti doesnot have very good mechanical properties, alloying is used to improve its performance, thus TiAlV, and TiAlNb are investigated and selected [8]. More recently, a comparison between TiZr and Ti [9] in terms of tensile strength, removal torque values (RTV), bone-to-implant contact (BIC), bone level change, and implant success rates indicates TiZr as a good alternative for Ti.Among TiZr alloys subjected to investigations, the alloys with higher Zr content [10,11] have a much larger passive range in the polarization curves and are found to be the most resistant to localized corrosion. Due to the deterioration of the mixture of oxide layer in the case of alloys with more than 50% Zr, our choice for coating TiZr alloy was Ti50Zr.

Zwitterionic coatings are an emerging surface modification for biomaterials that have demonstrated promising results as candidates for creating biofouling surfaces [12,13] at micro- and nanolevel. Amphoteric surfactant polymers such as poly(ethylene glycol) and polyurethane continue to be common materials used for anti-biofouling and drug release [14,15,16]. The major characteristics of these polymers responsible for their anti-biofouling abilities are firstly their electrical neutrality [17] enabling a reduced number of coulombic interactions with various charged domains of proteins, and secondly their hydrophilicity.

All zwitterionic coatings facilitate the formation of a hydration layer through hydrogen bonding between the surface of the material and the solvent, which acts not only as a physical barrier but also as an energetic barrier that the proteins have to overcome before adsorbing onto the surface [18]. Zwitterions, composed of an equal number of opposite charges in proximity, preserve net charge neutrality, can enhance biocompatibility, reduce fouling, have undergone many iterations in the last decade, and are recognized as the next-generation antifouling material [19,20]. Recently, an antibacterial surface coating based on a cysteine ligand covalently coupled via a spacer to a carboxylic backbone layer on an acrylic acid grafted silicone surface was developed [21]. The zwitterion coatings have followed two approaches: one focused on biocompatible materials and the other on more general anti fouling aspects at interfaces. Even though biocompatibility aspects are usually linked to nonbiofouling, the two concepts are not synonymous:biocompatibility, implying in vivo applications, has more requirements than simple nonadhesion. Conversely, biocompatibility does not necessarily mean nonfouling, and many metallic biomaterials such as titanium alloys adsorb proteins, are biocompatible and tolerate fouling for various periods.

As an alternative candidate for surface polymer coating, cysteine is a good selection because it has a small size, is a highly zwitterionic molecule at physiological pH, is biocompatible, isrelatively easily to fabricate and has established thiol chemistry. Cysteine is a crystalline proteinogenic amino acid, contributing to building proteins and has the formula C_6_H_12_O_4_N_2_S_2_. As a drug, cysteine exhibits important anti-inflammatory effects in human coronary arterial endothelial cells [22]. Surface functionalization of silica [23] and gold nanoparticles [24] was investigated to reach a Low-Fouling Zwitterionic Surface. The cysteine drug was proved to be biocompatible, to decrease protein adsorption and reduce biofouling significantly. Reducing postsurgical healing time of a biofunctionalized metallic implant with local drug immobilization to avoid bacterial contamination was successfully used both in vitro and in vivo [25]. These coatings demonstrate important fouling resistance in bioliquids as pure serum [26] and bacterial adhesion inhibition [27]. Our paper proposes a combined procedure of dual functionalization involving a silanization protocol and a cysteine immobilization on Ti50Zr alloy. As a novelty, this manuscript is an endeavor to correlate antibacterial, biocompatibility, and stability properties of implantable Ti50Zr covered with zwitterionic cysteine drug. It was demonstrated that the coating enhanced the production of intracellular ROS over time, which subsequently caused a significant increase in antibacterial index.

## 2. Materials and Methods

### 2.1. Materials and Reagents

Natrium hydroxide (NaOH), 3-aminopropil triethoxysilane (APTES), 3-maleimidopropionic acid *N*-hydroxysuccinimideesterand, and l-cysteine (Cys) were purchased from Sigma Aldrich (St. Louis, MO, USA) and used without further purification. Acetonitrile was obtained from Carlo Erba (Val de Reuil, France). Purified deionized water was obtained with a MilliQ-system (Billerica, MA, USA).

### 2.2. Substrate Coating Protocol

Ti50Zr plates were polished with SiC abrasive paper with different granulations (P 400, P 800, P1200; Carbimet, Lake Bluff, IL, USA). The plates were cleaned by sequential sonication inpurified water, ethanol and acetone, 15 min each.

Firstly, the Ti50Zr samples were subjected to NaOH treatment (10 M solution), 1 h at 60 °C. Resulted samples after this step were named Ti50Zr–OH. Then, samples were immersed in 3% APTES in acetone 1 h at room temperature, rinsed with acetone, followed by 30 min treatment in oven at 80 °C. Resulted samples were named Ti50Zr–OH+Si. In next step, plates were immersed in 6 mg/mL 3-maleimidopropionic acid *N*-hydroxysuccinimide ester in acetonitrile solution used as a linker. Immersion time was 1.5 h at room temperature. Then, they were washed with acetonitrile and water. Resulted samples were named Ti50Zr–OH+Si+Ester. In the last step, samples were incubated in a 2 mM purified aqueous solution of L-cysteine for 2 h. Finally, they were rinsed with purified water. Resulted samples were named Ti50Zr-cys. To determine the amount of cysteine bounded to the surface the UV-Vis spectrum of cysteine was recorded and a standard calibration curve was plotted. The quantity of cysteine detected in solution after TiZr immersion was 7.21 µg/mL, demonstrating that 70.24% of the amount of cysteine was adsorbed on the sample surface.

### 2.3. Surface Characterizations

Perkin Elmer Spectrum 100 FT-IR spectrophotometer (Shelton, DC, USA) was used to record ATR/FT-IR spectra to put in evidence the groups on the surface after each treatment step. The wave number domain was between 4000 and 600 cm^−1^. Presented spectra are the average of four scans with 4 cm^−1^ resolution.

SEM images of Ti50Zr-cys sample were recorded with Thermo Scientific FEI Quanta 650 FEG (Hillsboro, OR, USA) variable-pressure and environmental high-performance scanning electron microscope at 10 kV in high vacuum.

The surface wettability of the samples was recorded using Optical Contact Angle and Surface Tension Meter CAM100 (KSV Instruments, Espoo, FIN). Measurements were carried out with a Hamilton syringe, making water droplets of about 3–5 μL. Minimum three determinations were performed for each sample and presented values are the mean value. Experiments were made at room temperature and Microsoft Excel was used to calculate standard deviation.

### 2.4. Electrochemical Tests

All measurementswere performed using a potentiostat/galvanostat (Autolab 302 N) with NOVA specific software, in a three-electrode electrochemical cell. Ti50Zr samples were mounted on a working electrode and 1 cm^2^ of sample surface was exposed to the electrolyte (NaCl 0.9% aqueous solution). A Pt rod (Metrohm, Herisau, Switzerland) was used as counter electrode and Ag/AgCl 3 M KCl (Metrohm) was used as reference electrode.

Open circuit potential (OCP) measurements were performed for 1 h at room temperature. Tafel plots were recorded at initial immersion time, 24, 48 and 72 h after immersion and were registered using 2 mV/s scan rate, at ±260 mV vs. OCP. Electrochemical impedance spectroscopy (EIS) measurements were recorded at OCP in a frequency domain between 10^5^ Hz and 10^−2^ Hz. Experiments were performed with 10 mV AC potential amplitude.

### 2.5. In Vitro Biocompatibility Assessment

In vitro cell response to uncoated Ti50Zr alloy and silane-cysteine coatings were analyzed using *HGF-1* gingival fibroblasts cell line (purchased from American Type Culture Collection (ATCC), Cat. No. CRL-2014, Rockville, MD, USA). The cells were cultured in Dulbecco’s Modified Eagle Medium (DMEM; Gibco/Invitrogen, Carlsbad, CA, USA) with an addition of 10% fetal bovine serum (FBS; Gibco/Invitrogen, Carlsbad, CA, USA) at 37 °C in a humidified atmosphere with 5% CO_2_. After 48 h of cell exposure to sterilized samples, several biocompatibility tests were performed. The lactate dehydrogenase (LDH) amount released in culture medium was determined as a measure of cell membrane integrity and cell viability using a commercial kit (Cytotoxicity Detection Kit-LDH, Roche, Basel, Switzerland) by reading the absorbance at 490 nm using a FlexStation 3 microplate reader (Molecular Devices, San Jose, CA, USA). In addition, the level of nitric oxide (NO) released in the culture medium as an indicator of inflammation was assessed using the Griess reagent (a stoichiometric solution (*v*/*v*) of 0.1% naphthylethylendiamine dihydrochloride and 1% sulphanilamide in 5% H_3_PO_4_) after reading the absorbance at 550 nm. The intracellular ROS level was assessed using a fluorescent compound 2′,7′-dichlorofluorescein diacetate (DCFH-DA, Sigma-Aldrich, St. Louis, MO, USA). The fibroblasts were washed with PBS and incubated with the dye for 30 min at 37 °C. The fluorescence was quantified using a fluorimeter (FP-750 Spectrofluorometer, Jasco, Tokyo, Japan) with an excitation wavelength of 488 nm and emission wavelength of515 nm. All results were expressed relative to control after fluorescence intensity was reported to the number of viable cells of each sample. The cell cytoskeleton morphology was visualized via fluorescence imaging using cells fixed with 4% paraformaldehyde for 20 min and permeabilized with 0.1% Triton X-100% bovine serum albumin for 1 h. Filamentous actin (F-actin) was labeled with 20 μg/mL phalloidin conjugated with fluorescein isothiocyanate (FITC) (Sigma-Aldrich, Munich, Germany) and nuclei were counterstained with 2 μg/mL 4′,6-diamidino-2-phenylindole (DAPI) (Molecular Probes, Life Technologies, Carlsbad, CA, USA). Images were captured using a fluorescence microscope Olympus IX71 (Olympus, Tokyo, Japan).

All data are expressed as mean value ± SD of three independent experiments. Statistical differences between samples and control were evaluated by Student’s *t*-test (Microsoft Excel, 2010 version) and a value of *p* < 0.05 was considered statistically significant. 

### 2.6. Antibacterial Effect

Antimicrobial activities of samples were appraised against *Escherichia coli* (ATCC 25922), which is a Gram-negative, coliform bacterium, and *Staphylococcus aureus* ATCC 6538, a common causative Gram-positive pathogen. The measurements were performed using the turbidimetric method. Antibacterial test was routinely cultured in Columbia Agar with 5% sheep blood (Oxoid) for 24 h at 37 °C. Then, a bacterial suspension (inoculum) was prepared with sterile physiological solution, and adjusted with a densitometer to a MacFarland 0.5 up to a CFU countto 10^8^ CFU/mL.

The three tested samples, uncoated, with adsorbed material and the bonded were immersed in the bacterial suspension in sterile polypropilene tubes. As a negative control, sterile saline solution was used, and, as positive control, the inoculum. All recipients were incubated at 37 °C for 24 h. Antimicrobial efficacity test was performed by reading absorbance of microbial cultures at 600 nm with an automated analyzer Chemwell 6010 and the antibacterial activity was calculated using Equation (1):

(1) $$I\%=\frac{\left(C_{24}-C_{0}\right)-\left(T_{24}-T_{0}\right)}{\left(C_{24}-C_{0}\right)}\times 100$$

where *I* is the growth inhibition index; *C*_0_ is the blank-corrected optical density of the positive control at time 0; *C*_24_ is the blank-corrected optical density of the positive control after 24 h; *T*_0_ is the blank-corrected optical density of infected media in the presence of test samples; and *T*_24_ is the blank-corrected optical density of infected media in the presence of test samples at 24 h.

## 3. Results and Discussions

### 3.1. Surface Characterization

**Figure 1:**
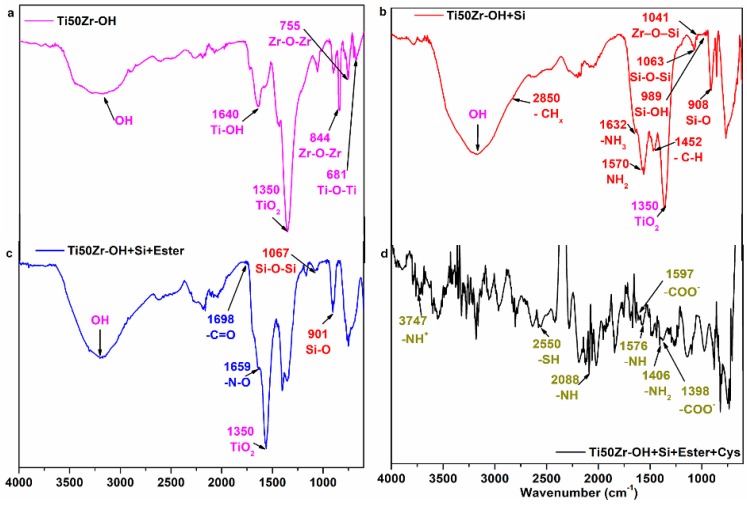

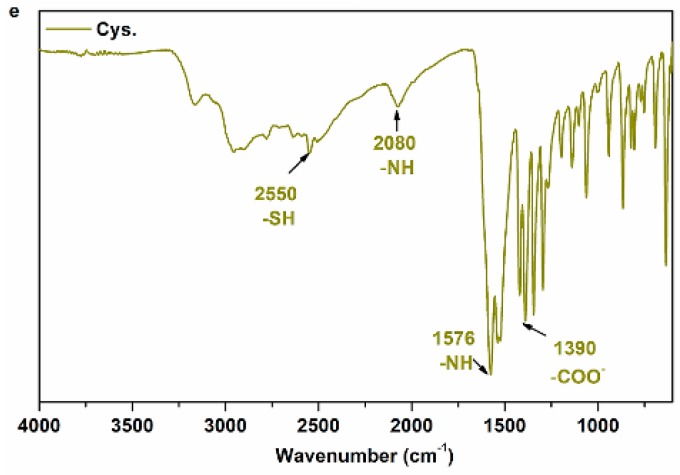
FTIR spectra of: (**a**) Ti50Zr substrate after NaOH treatment; (**b**) Ti50Zr–OH+Si; (**c**) Ti50Zr–OH+Si+Ester; (**d**) Ti50Zr-cys; and (**e**) Cys.

The FT-IR spectrum for Ti50Zr–OH sample, obtained after first coating step, revealed the success of the treatment by the stretch vibration of –OH, corresponding to the strong peak between 3000 and 3500 cm^−1^ (Figure 1a). The absorption band at 1640 cm^−1^ was assigned to bending vibration of coordinated H_2_O as well as Ti–OH. Oxides corresponding to the substrateand Ti50Zr are present. Titanium presence is revealed by the sharp peak at 1351 cm^−1^, attributed to the lattice vibrations of TiO_2_ and the peak at 688 cm^−1^, assigned to the stretching of Ti–O–Ti [27]. The peak at 844 cm^−1^ corresponds to Zr–O bond and the sharp peak at 755 cm^−1^ corresponds to Zr–O–Zr bond, showing the presence of zirconium [28].

The obtained spectra for Ti50Zr–OH+Si sample are shown in Figure 1b. The peaks at 2850 cm^−1^ and 1452 cm^−1^ can be attributed to the stretch and respective bending vibrations of C–H bond from APTES [29]. The peak at 1570 cm^−1^ may be due to the N–H bending vibration of the NH_2_ terminal group of APTES. The peak at 1632 cm^−1^ corresponds to the asymmetric –NH_3_ deformation mode [30]. Existence of Zr–O–Si is indicated by the peak at 1041 cm^−1^ [29]. The peak at 1028 cm^−1^ can be attributed to Si–O–Si [30]. Because of the partial hydrolysis of APS, a peak at 908 cm^−1^ is visible and could be assigned to Si–O stretching of SiOH [4]. APTES absorption onto the Ti50Zr–OH was also confirmed by peaks assigned to the Si–O–H found at ~1105 cm^−1^ and Si–O–Si groups at~1063 and ~ 990 cm^−1^ [31].

After Ti50Zr–OH+Siwas exposedto the linker (3-maleimidopropionic acid *N*-hydroxysuccinimide ester), the obtained FTIR spectra (Figure 1c) revealed that the ester has a characteristic peak at ~1730 cm^−1^ [32]. The ester presence on the surface is also shown by the two peaks, at 1660 cm^−1^ attributed to the N–O bond and 1700 cm^−1^ attributed to the C=O bond [33].

Figure 1d shows the FTIR spectra of Ti50Zr-cys sample and Figure 1e corresponds to commercial l-Cysteine. The stretching vibrations of S–H bond are indicated by the peak at 2550 cm^−1^ in both spectra. Cysteine presence is also revealed by peaks at 2088 cm^−1^ for the sample and 2080 cm^−1^ for commercial l-cysteine. They were assigned to the stretching vibrations of the N–H [34]. Peaks at 1597 and 1398 cm^−1^ for coated samples and 1390 cm^−1^ in the case of commercial l-Cysteine were attributed to the asymmetric and symmetric stretching of COO–N–H bending. In the spectra of Ti50Zr-cys sample, the peaks at1624, 1597 and 1406 cm^−1^ correspond to asymmetric and symmetric stretching of COO– and –H_2_ [35].

**Figure 2:**
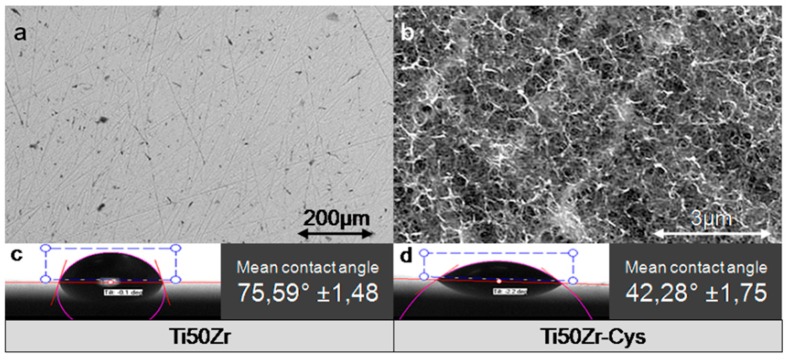
SEM images (**a**,**b**); and water contact angle images (**c**,**d**) ofTi50Zr substrate and Ti50Zr-cys sample.

In the SEM image presented in Figure 2a, some scratches are visible on the Ti50Zr substrate left behind after polishing. For Ti50Zr-cys sample (Figure 2b), a thin, webbed layer is visible on the surface. The formed tendril-like cysteine structures vary in length, but their thickness is around 50 ± 8 nm as a mean from 10 measurements.

The mean water contact angle values are 75.59° ± 1.48° for Ti50Zr substrate and 42.28° ± 1.75° for Ti50Zr-cys sample. With this coating, the almost hydrophobic substrate became hydrophilic.

### 3.2. Electrochemical Tests 

For the Ti50Zr substrate, OCP value at initial immersion time was −0.51 V. During the first hour of immersion, an oxide layer was formed and the OCP values became more electropositive (−0.308 V). In time, the thickness of the oxide layer lightly increased and the OCP reached values close to 0 V after 72 h of immersion.

For Ti50Zr-cys samples, the initial OCP values (−0.24 V) have insignificant variations in time, and were electropositive compared to substrate.

**Figure 3:**
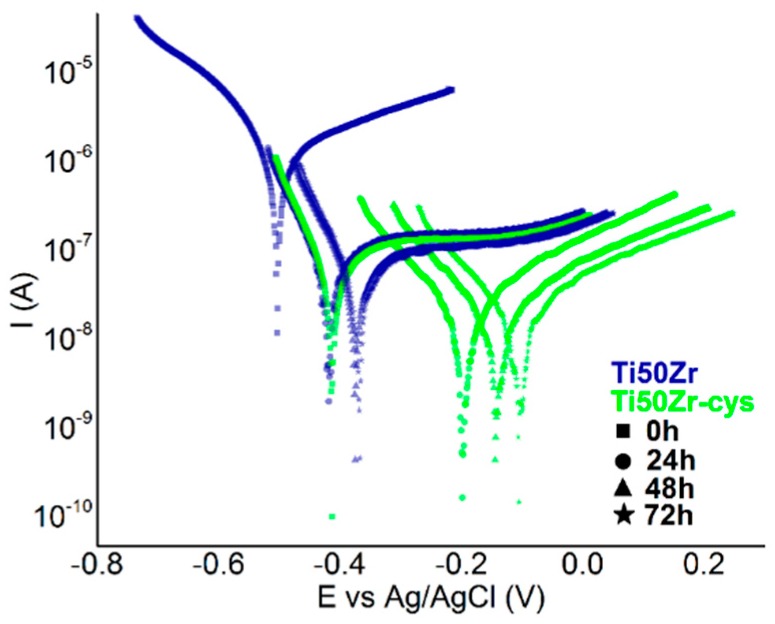
Tafel plots in time for Ti50Zr substrate and coated samples.

Tafel plots obtained during immersion in NaCl 0.9% are presented in Figure 3. It is visible that, in the beginning, the corrosion potential for the substrate has more electronegative values compared to Ti50Zr-cys sample and the corrosion currents have lower values for all coated samples.

Data obtained with Nova Software are presented in Table 1.

The treatment performed on the Ti50Zr samples does not affect the electrochemical stability, all coated samples being stable during the immersion. The calculated corrosion speeds show that the coated samples can be classified to perfectly stable materials according to the Standard resistance classes ISO 8044/2000.

**Figure 4:**
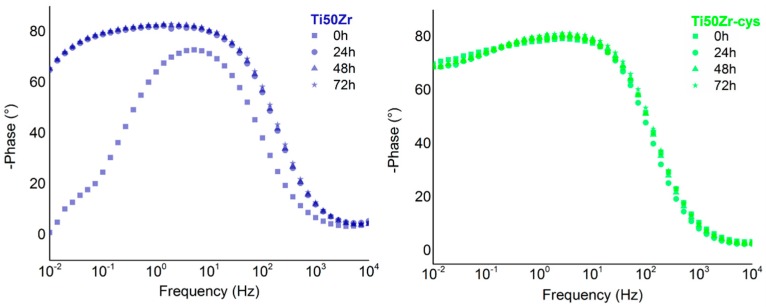
Bode phase plots in time for Ti50Zr substrate and coated samples.

Experimental data obtained from EIS measurements are presented in Figure 4 as Bode phase plots. For Ti50Zr at initial immersion time, the phase angle at medium frequencies reaches 67° and decreases towards 0° for low frequencies. This hints that the natural oxide layer is thin. In time, the phase angles increase at all frequencies, having a pseudocapacitive behavior at medium and lower frequencies corresponding to the growth of the passive mixt oxide film.

All coated samples have similar phase angles regardless of immersion time or frequencies. This behavior shows a great stability of the coated samples.

**Figure 5:**
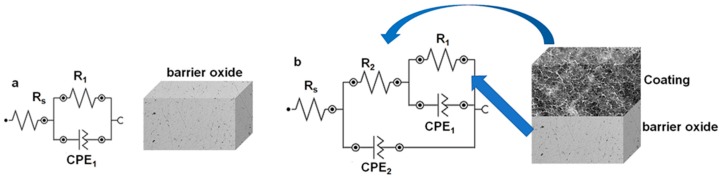
Equivalent circuits used to fit EIS data: (**a**) for Ti50Zr substrate; and (**b**) coated samples.

Data were fitted with Nova Software using the equivalent circuits presented in Figure 5. For Ti50Zr substrate, a Randles simple circuit was proposed. The circuit is composed of a resistance element attributed to the solution (*R*_s_) linked in series with two parallel elements: a resistance (*R*_1_) and a Constant Phase Element (CPE_1_) representing the interface between the electrolyte and the Ti50Zr substrate. For the coated samples, a second resistance (*R*_2_) and a second Constant Phase Element (CPE_2_) were inserted to the previous circuit between (*R*_s_) and (R1-CPE1) to represent the coating present on the surface.

The electrical parameters obtained from EIS are presented in Table 2. χ^2^ exhibited values on the order of 10^−2^ to 10^−3^, which guarantees that the circuit was adequately fit. All samples show pseudocapacitive behaviors.

### 3.3. In Vitro Biocompatibility

Zwitterionic functional groups of cysteine, which support its functionality to reduce or eliminate non-specific adsorption to the solid/liquid interface, have already been presented in the literature [19]. The antifouling mechanism of zwitterionic compounds is generally attributed to the presence of a hydration layer on the surface. The zwitterion allows the H-bonding organization to remain unperturbed, while the single charge reorients the waters to a more disordered structure and less H-bonded state [36]. Zheng et al. demonstrated with simulations that a layer of bonded water molecules adjacent to hydrogen-bonding acceptors is responsible for reducing protein fouling [37]. By forming a strong hydration shell, coated surfaces demonstrate a decreased interaction with the biological environment.

**Figure 6:**
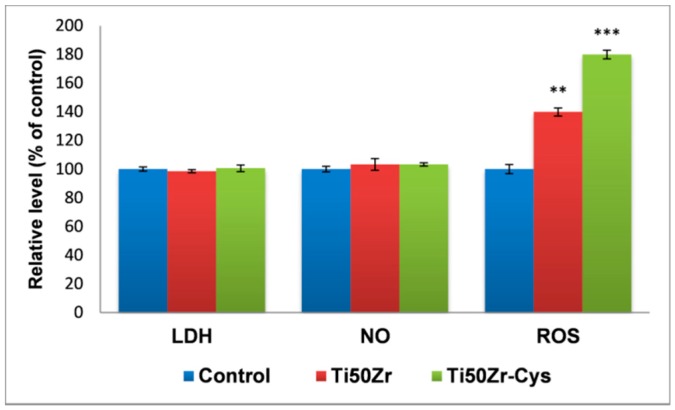
Cellular response revealed by LDH and NO release from gingival fibroblasts and the intracellular level of reactive oxygen species after 48 h exposure of HGF-1 gingival fibroblasts to cysteine coated Ti50Zr alloys. Data are expressed as mean ± standard deviation (*n* = 3) and shown relative to control (untreated cells) (** *p* < 0.01 and *** *p* < 0.001).

The influence of cysteine coating of Ti50Zr alloys on cell viability and membrane integrity, as well as their potential to generate an inflammatory response, were evaluated by in vitro tests performed on normal human gingival fibroblasts, as presented in Figure 6.

Our results show that 48 h exposure of fibroblasts to cysteine treated samples did not cause significant changes in cell viability. Because LDH and NO levels released in culture media were very close to the control values, the present study demonstrates that the cysteine-coated did not induce a significant inflammatory response.

Different parameters may be responsible for the cytotoxicity of biomaterials, but the ability to generate reactive oxygen species (ROS) and oxidative stress is the main mechanism to produce oxidative lesions in biomolecules. Oxidative stress occurs as a result of an imbalance between the rate of ROS formation and the antioxidant capacity of the cell to neutralize those [38]. Therefore, to assess the potential of cysteine-treated Ti50Zr alloys to induce oxidative stress in gingival fibroblasts, the intracellular ROS level was measured using the fluorescence intensity of dichlorofluorescein (DCF). As shown in Figure 6, exposure of gingival fibroblasts to cysteine-coated Ti50Zr alloys generated a similar ROS increase by 80% relative to control. Surface topography and mechanical stress could be the main factors that stimulate ROS production. We might consider that the reactive species rather acted as modulators of the key intracellular signaling pathways [39].

Even if intracellular ROS concentrations were high enough in the gingival fibroblasts exposed to cysteine-coated Ti50Zr alloys, the cellular antioxidant defense system was probably effective and succeeded to counteract partially the oxidative stress. Actin filaments represent the major component of the cellular cytoskeleton, forming links or three-dimensional networks [40]. Assembling and disassembling of actin microfilaments, their crosslinking in bundles or networks, and interaction with other cellular components (the plasma membrane) providing mechanical support determine the shape of cells and allow cell surface movement, migration and division, as well as phagocytosis of different particles [41].

**Figure 7:**
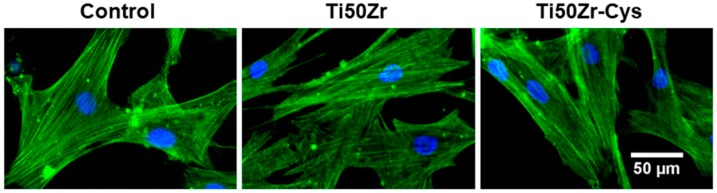
The actin cytoskeleton organization of HGF-1 gingival fibroblasts after 48 h of cultivation on cysteine coated Ti50Zr alloys. Scale bar: 50 µm.

Dynamic changes of the actin cytoskeleton evidenced by the fluorescence microscopy (Figure 7) were consistent with the results of the biocompatibility tests presented in Figure 6. Thus, it was observed that the cells maintained their specific elongated morphology and established numerous focal adhesions after 48 h of exposure, which confirmed that cysteine coatings on Ti50Zr alloys did not affect the behavior of gingival fibroblasts. These modified surfaces were harmless to the gingival cells, proving a good biocompatibility. The preservation of cell viability is well correlated with the decrease of contact angle determining better hydrophilicity generally preferred by cell.

### 3.4. Antibacterial Effect

The bacterial growth inhibition ratio (I%) is presented in Table 3 for both tested bacteria, *S. aureus* and *E. coli*, before and after the coating steps, showing that the presence of the zwitterionic cysteine drug coating highly increases the antibacterial activity of the samples.

**Figure 8:**
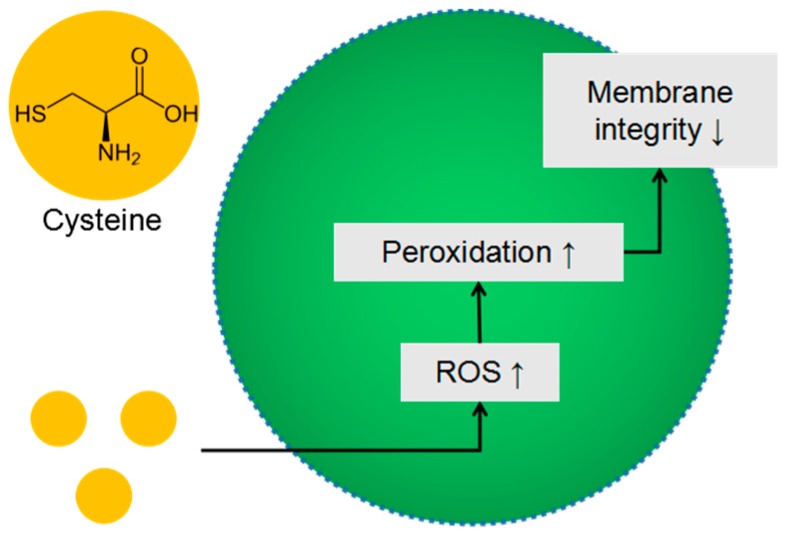
Schematic model of zwitterionic cysteine drug in antibacterial action.

As expected, the bacterial inhibition level for *S. aureus* is slightly smaller than the value for *E.coli* because Gram-positive bacteria are more receptive to drugs than Gram-negative bacteria, due to the absence of the outer membrane. This antibacterial activity is correlated with the increase of ROS production presented in Figure 6 and in the literature for the action of other drugs as well [38]. In the *E. coli* case, cysteine toxicity has been suggested to be due to the inhibition of RNA synthesis as well as to a cysteine-specific inhibition of the branched-chain amino acid synthesis [42,43]. Another mechanism described to inhibit the growth of Gram-negative bacteria is that cysteine undergoes a metal ion-catalyzed autoxidation, leading to hydrogen peroxide production [44]. However, in the case of *S. aureus*, one of the most important enzymes produced by this microorganism is a protective enzyme called catalase [45] that is responsible for the conversion of hydrogen peroxide to water and molecular oxygen. As the bacterial membrane is composed of phospholipid bilayer, the production of ROS prior to cysteine treatment might oxidize the lipid content on the cell membrane, hence affecting the bacterial membrane integrity [46]. The cell envelope of the prokaryotic cells contains proteins stabilized by one or more disulfide bonds [47]. A disulfide bond can serve structural, catalytic and signaling roles. Due to the relatively high concentration of external cysteine, the thiol group could attack the disulfide bond of proteins that affect the tridimensional configuration of protein and could disorganize the cellular envelope generating osmotic shock and bacterial death. Such an empiric model of the ROS role in antibacterial effect is presented in Figure 8 and can be integrated in the well-known mechanism of antibacterial activity. Such model in three stages could be integrated into the already accepted general mechanisms of bacterial interactions with support material [48].

The general mechanism involves an instantaneous and reversible physical first phase, and a time-dependent irreversible molecular and cellular second phase [49]. At the contact with a tissue, a competition between bacterial colonization and tissue integration takes place. The accumulated biomass of bacteria and their extracellular material on various support surface (in our study, adsorbed and bonded material) forms what is called biofilm, which protects bacteria from detachment. Biofilm formation is a part of physical stage starting with bacterial adhesion to surfaces after the initial attraction of the cells to the surface, adsorption and attachment. Cells adherence is very important in biofilm formation, but even for good adherence, some cells escape from the extra cellular material and initiate new colonization sites; the surface properties of the substrate play an important role in determining the final result. Zwitterionic drugs, such as cysteine, can avoid extended colonization because ofthe presence of the hydration layer on the surface [33]. Such behavior aspects have been discussed on commercially pure titanium and zirconium oxide [50].

## 4. Conclusions

Using SEM, FT-IR, electrochemical techniques, and contact angle determinations, the structure, hydrophilic balance and stability properties of a zwitterionic cysteine coating was investigated. The infrared structure evidenced large bands due to hydroxylic groups which formed a hydration layer and determined the decrease of contact angle. This layer is responsible for antibacterial effect as well. The cell response to cysteine coating determined with gingival fibroblasts measuring lactate dehydrogenase activity, concentrations of nitric oxide and intracellular level of reactive oxygen species indicated that the coating is biocompatible, and no cytotoxicity was evidenced. Having more stability in time, increased antibacterial effect due to enhance production of intracellular reactive oxygen species over time and biocompatibility, cysteine coating revealed better bioperformance than uncoated alloy. This coating is a promising candidate for reducing postsurgical time of implantable Ti50Zr alloy.

## Figures and Tables

**Table 1:** The electrochemical stability data obtained from Tafel plots.

Immersion Time (h)	Sample	*E*_corr_ (V)	*J*_corr_ (A/cm^2^)	Corrosion Rate (mm/year)
0	Ti50Zr	−0.504	1.361 × 10^−6^	131.1 × 10^−4^
	Ti50Zr-cys	−0.417	4.048 × 10^−8^	3.898 × 10^−4^
24	Ti50Zr	−0.413	3.712 × 10^−8^	3.574 × 10^−4^
	Ti50Zr-cys	−0.198	1.401 × 10^−8^	1.349 × 10^−4^
48	Ti50Zr	−0.374	3.481 × 10^−8^	3.322 × 10^−4^
	Ti50Zr-cys	−0.144	1.450 × 10^−8^	1.396 × 10^−4^
72	Ti50Zr	−0.366	3.182 × 10^−8^	3.064 × 10^−4^
	Ti50Zr-cys	−0.105	1.400 × 10^−8^	1.348 × 10^−4^

**Table 2:** The fitting values of the equivalent circuit elements for substrate and coated samples.

Immersion Time (h)	Sample	Rs (Ω)	R2 (Ω) ×10^−5^	CPE2		R1 (Ω) ×10^−6^	CPE1		χ^2^
				Yo1 (S × sn) ×10^5^	N2		Yo2 (S × sn) ×10^5^	N1	
0	Ti50Zr	63.79	-	-	-	0.01	5.22	0.89	0.05
	Ti50Zr-cys	52.74	1.23	3.48	0.91	1.40	1.18	0.83	0.01
24	Ti50Zr	58.73	-	-	-	1.32	2.47	0.92	0.02
	Ti50Zr-cys	66.87	1.64	3.06	0.92	3.77	1.42	0.85	0.01
48	Ti50Zr	61.53	-	-	-	1.49	2.23	0.92	0.02
	Ti50Zr-cys	60.92	1.40	2.87	0.93	4.09	1.18	0.89	0.007
72	Ti50Zr	61.62	-	-	-	1.59	2.08	0.92	0.02
	Ti50Zr-cys	59.03	1.10	2.61	0.94	4.45	1.05	0.90	0.004

**Table 3:** The bacterial inhibition level of zwitterionic coating.

Sample	Ti50Zr	TiZr-cys
I% *S. aureus*	27.39 ± 0.15	56.74 ± 0.28
I% *E. coli*	29.15 ± 0.23	63.90 ± 0.33
